# Seroprevalence of hepatitis B and C virus infection among patients attending serology laboratory of Gondar University Hospital

**DOI:** 10.1186/1756-0500-6-164

**Published:** 2013-04-25

**Authors:** Habtie Tesfa, Belete Biadgo, Fasika Getachew, Kifle Tegegne, Gizachew Yismaw, Dagnachew Muluye

**Affiliations:** 1 School of Biomedical and Laboratory Sciences, College of Medicine and Health Sciences, University of Gondar, P.O.Box. 196, Gondar, Ethiopia

**Keywords:** Hepatitis B virus, Hepatitis C virus, Prevalence, Ethiopia

## Abstract

**Background:**

Hepatitis B virus (HBV) and hepatitis C virus (HBV) infections accounts for substantial proportions of the world wide liver disease. The two hepatotropic virus share common modes of transmission and their co-infection are common. Patients with dual HBV and HCV infection have more severe liver disease and are at increased risk of progression to hepatic cell carcinoma. This study was aimed to assess the prevalence of HBV and HCV among patients attending serology laboratory of Gondar University Teaching Hospital.

**Methods:**

A retrospective study was conducted from January 2007 to December 2011 at serology laboratory of Gondar University Teaching Hospital. Data were cross checked for completeness, entered and analyzed using SPSS version 16 statistical package.

**Results:**

From the total of 2,684 clinicaly suspected hepatitis patients, 563 (20.98%) were positive for HBsAg and anti-HCV test. Of all, 14.4% were seropositive for HBV (male= 7.89% female=6.27%) (p-value=0.011) while 12.4% were seropositive for HCV (male =7.6% and female=4.13%) (p-value<0.001). The co-incidence of HBV and HCV were found to be 36 (6.39%) (male=3.9% and female=2.4%) (p-value <0.001). Majority of HBV (30.2%) and HCV (30.7%) infections were detected among age group of 25–34 years old (p-value=0.36) and 15–24 years old (p-value<0.001) respectively.

**Conclusion:**

The overall prevalence of HBV and HCV is high. Males and younger age groups were more affected. Community awareness about the transmission and prevention of viral hepatitis infection should be strengthened by giving health education and herd immunization.

## Background

Infection with hepatitis B virus (HBV) and hepatitis C virus (HCV) affects the liver and results in a broad spectrum of disease outcomes [1]. Hepatitis B virus is a member of the family Hepadnaviridae which contain a unique deoxyribonucleic acid (DNA) genome virus while HCV is a member of family Flaviviridae under RNA virus. An infection with HBV may spontaneously resolve and lead to protective immunity, chronic infection and, in rare cases, acute liver failure with a high risk of death. In contrast to HBV, an infection with HCV becomes chronic in most cases. People with chronic HBV and/or HCV infection remain infectious to others and are at risk of serious liver disease such as liver cirrhosis or hepatic cell carcinoma (HCC) later in life [2,3].

Approximately one third of the world’s population has been exposed to HBV and an estimated 350 million people are chronically infected [3,4]. Nearly 350 million individuals has been infected with HBV and each year, an estimated 1 million persons die from chronic complications of the disease. Although chronic HBV infection has a worldwide distribution, the vast majority of infected persons reside in Asia, the Middle East and Africa [5]. It has been estimated that, annually, about 1.2 million people are dying globally from chronic HBV infection, cirrhosis and liver cancer [6].

The World Health Organization (WHO) estimated that 3% of the world’s population are infected with HCV, resulting in a total of 120 to 170 million people [7,8]. There is a distinct geographical variation in both HBV and HCV prevalence and incidence due to lack of proper health facilities, poor economical status and less public awareness about the transmission of major communicable diseases [9].

Suppression of HBV replication by HCV in acutely or chronically infected patients is well-described phenomenon. In vivo study, in chimpanzees, showed that acute HCV super infection in chronic HBV infection resulted in marked reduction in the titer of serum HBsAg [10,11].

Both HBV and HCV share similar modes of transmission and co-infection with the two viruses is common, especially in areas with a high prevalence of HBV infection and among people at high risk of parenteral infection [12]. Hepatitis B virus and hepatitis C virus can be transmitted by transfusion of infected blood and carrying out healthcare procedures using contaminated instruments and other unsafe practices. Perinatal and sexual exposures to HBV are also highly efficient modes of transmission [13]. Patients with dual HBV and HCV infection have more severe liver disease, and are at increased risk of progression to HCC [12]. Chronic infection develops in the majority of people infected with HBV early in life while the majority of people infected with HBV as adults will recover completely [3].

Generally, patients who are clinically suspected for HBV and HCV have been consistently shown to have high prevalence of infection. Knowledge about the situation of hepatitis infection in developing countries like Ethiopia is limited. Awareness about the seriousness of this condition is limited among the care takers. Thus this study was conducted to determine extent of hepatitis infection among patients attending serology laboratory of Gondar University Teaching Hospital.

## Method

### Study design and area

A retrospective study was conducted among clinically suspected hepatitis patients attending serology laboratory of Gondar University Teaching Hospital from January 2007 to December 2011. Gondar University Teaching Hospital is situated in Gondar Town, located 747 km away from Addis Ababa, capital city of Ethiopia. It acts as referral centre for four district hospitals in the area. It has a range of specialties including pediatrics, surgery, gynecology, psychiatry, HIV care and an outpatient’s clinic and serves a population of more than five million across the region. As a teaching hospital, it plays an important role in teaching, research and community service.

The study populations were all patients who gave blood and tested for HBV and HCV at Gondar University Teaching Hospital from January 2007 to December 2011.

Data were collected from log book of serology laboratory of Gondar University Teaching Hospital after checking the completeness of patients’ data. Data were entered and analyzed by using SPSS version 16 statistical software. Differences in proportions were evaluated by Pearson’s chi-square and p<0.05 were considered to be statistically significant.

Data were collected after ethical clearance obtained from the School of Biomedical and Laboratory Sciences, College of Medicine and Health Science, University of Gondar. Permission was obtained from the Head of serology laboratory, Gondar University Teaching Hospital before the data collection.

## Result

A total of 2,684 serologically tested patients were included. Of these, 1,373 (51.2%) were females and 1,311 (48.8%) were males. The majority of study subjects 1,541 (57.4%) were aged from 15–34 years old [Table 1]. The overall prevalence of HBV and HCV was 14.6% and 12.41% respectively. There was significant difference among gender with p value of 0.011 for HBV (male=7.9%, female=6.3%) and 0.001 for HCV (male =7.6%, female 4.13%). The majority of infected individuals were aged 15–34 years old for both HBV and HCV infection with p-value 0.36 and <0.001 respectively.

**Table 1:** Socio-demographic characteristics of study subjects at serology laboratory of Gondar University teaching hospital from January 2007 to December 2011

**Variables**	**Frequency (n)**	**Percentage (%)**
**Sex**		
Male	1311	48.8
Female	1373	51.2
**Age**		
0-14	335	12.5
15-24	768	28.6
25-34	773	28.8
35-44	424	15.8
45-54	212	7.9
55-64	99	3.7
≥65	73	2.7

**Figure 1:**
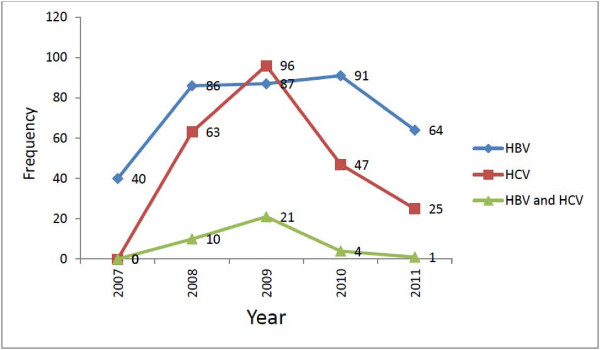
Trend prevalence of HBV, HCV and HBV-HCV co-infection at serology laboratory of Gondar University Teaching Hospital from January 2007 to December 2011.

HBV was found to be more prevalent among study subjects aged 25–34 years old (30.2%) and HCV was more prevalent among those aged 15–24 years old (30.7%). Among seropositve study subjects, 251 (18.28%) were females and 348 (6.25%) were males. The prevalence of co-infection was comparatively high in males. Of HBV and HCV mixed infected 36 (2%) study subjects, 1.3% were males and 0.7% were females (p-value<0.001) [Table 2]. The majority of co-infected study subjects were 15–24 years old 27.8% (p-value<0.001). The prevalence of HBV and HCV was relatively fluctuating from year to year. The prevalence of HBsAg was 1.5% in 2007, 3.2% in 2008 and 2009, 3.4% in 2010 and 2.4% in 2011. The anti-HCV prevalence was 0.0% in 2007, 2.3% in 2008, 3.6% in 2009, 1.8% in 2010 and 0.9% in 2011 [Figure 1].

**Table 2:** Percentage of HBV, HCV and HBV-HCV co-infection among study subjects at serology laboratory of Gondar University teaching Hospital from January 2007 to December 2011

**Variables**	**Total HBV suspected (%)**	**HBsAg positive (%)**	**α**^**2**^	**P-value**	**Total HCV suspected (%)**	**HCV positive (%)**	**α**^**2**^	**P-value**	**Total HBV-HCV suspected (%)**	**HBsAg and HCV positive (%)**	**α**^**2**^	**P-value**
**Sex**												
Male	1275(49.1)	205(55.7)	9.07	0.011	924(49.6)	143(61.9)	17.4	<0.001	672(47.3)	23(63.9)	5.4	<0.00
Female	1324(50.9)	163(44.3)			938(50.4)	88(38.1)			750(52.7)	13(36.1)		1
**Age**												
0-14	322	37(10.1)	13.1	0.36	222	18(7.8)	53.6	<0.001	177	4(11.1)	5.4	<0.00
15-24	742	90(24.5)			489	71(30.7)			374	10(27.8)		1
25-34	750	111(30.2)			536	58(25.1)			414	9(25.0)		
35-44	414	69(18.8)			311	40(17.3)			235	7(19.4)		
45-54	205	39(10.6)			171	23(10)			120	2(5.6)		
55-64	96	14(3.8)			77	18(7.8)			56	4(11.1)		
≥65	70	8(2.2)			56	3(1.3)			46	0 (0)		

## Discussion

HBV and HCV infections are serious health problems which affect approximately two billion and one hundred seventy million people across the world, respectively [7]. In this study the prevalence of HBV and HCV was 14.6% and 12.4% respectively. However, similar study conducted in Gondar, Mekele, Bahirdar and Addis Ababa among blood donors showed lower prevalence of HBV and HCV [14-16]. This inconsistency might be due to exclusion of donors with clinical sign and symptom of hepatitis for blood donation.

Moreover, a study conducted in Pakistan [17] showed 3.94% HBV infected males which is lower as compared to the present study (7.9%). Another similar study conducted in Pakistan surgical outpatient diseases indicated a prevalence of 8.6% HBsAg positve and 10.8% HCV positive among males [18]. This finding is almost similar to the present study. A study conducted in Egypt on intravenous drug addicts revealed 24% cases of HBsAg positive and 33% was positive for anti-HCV. This study was inconsistent with the present study which might be due to differences in study subjects who were highly vulnerable to hepatitis infection due to shared use of needle and more parenteral exposure [19].

Another study conducted in Sudan Maternity Hospital reported that 5.6% was positive for HBsAg [20]. This study was in line with the present finding (6.27%) on females. A prospective study conducted in Addis Ababa, Ethiopia revealed a total prevalence of 4.7% [21] HBV infected which is inconsistent with the present study (14.6%). This might be due to the difference in the study period, study design and study subjects.

Furthermore, a prospective study conducted in Pakistan showed 0.9% and 2.5% prevalence of HCV among females and males respectively [17]. This result is inconsistent with the present study which was 4.13% in females and 7.6% in males. This inconsistency might be due to variation in study population,-screening methods and study design.

Another study conducted in Nigeria revealed 8.3% HBV-HCV co-infection [16]. This finding is inconsistent with the current study which is 2%. A prospective study conducted in Burkina Faso showed prevalence of 29.4% HBsAg positive, 3.9% HCV positive and 2.2% HBV-HCV co-infection. The prevalence of HBV-HCV co-infection is in line with our study [22].

In this study we tried to provide insights of hepatitis virus prevalence which is not well studied in the area. Since the study is secondary data based finding; data incompleteness and poor document retention system were limitation of this study.

## Conclusion

The overall prevalence of HBV and HCV is higher in males and age group of 15–34 years old. The trend prevalence is still going constantly which may affect this productive age group especially males. It is recommended if immunization program among adults is implemented in the setup. Information about the transmission, prevention and awareness of viral hepatitis infection should be strengthened by giving health education and herd immunization.

## Abbreviations

HBsAg: Hepatitis B suface antigen; HBV: Hepatitis B virus; HCC: Hepatic cell carcinoma; HCV: Hepatitis C virus; HIV: Human immunodeficiency virus; WHO: World health organization

## Competing interests

The authors declare that they have no competing interests.

## Authors’ contributions

HA, BB, FG, KT, were the primary researcher, conceived the study, designed, participated in data collection; conducted data analysis drafted and finalized the manuscript for publication. GY and DM assisted in data collection and reviewed the initial and final drafts of the manuscript. HT, BB and DM interpreted the results, and reviewed the initial and final drafts of the manuscript. All authors read and approved the final manuscript.
